# Diabetes screening intervals based on risk stratification

**DOI:** 10.1186/s12902-016-0139-1

**Published:** 2016-11-22

**Authors:** Sachiko Ohde, Emily McFadden, Gautam A. Deshpande, Hiroshi Yokomichi, Osamu Takahashi, Tsuguya Fukui, Rafael Perera, Zentaro Yamagata

**Affiliations:** 1 Center for Clinical Epidemiology, St. Luke’s International University, 10-1 Akashi-cho, Chuo, Tokyo 104-0044 Japan; 2 Department of General Internal Medicine, St. Luke’s International Hospital, 9-1 Akashi-cho, Tokyo, 104-8560 Japan; 3 Department of Internal Medicine, University of Hawaii, Honolulu, Hawaii USA; 4 Nuffield Department of Primary Care Health Sciences, University of Oxford, Oxford, UK; 5 Department of Health Science, Basic Science for Clinical Medicine, Division of Medicine, Graduate School Department of Interdisciplinary Research, University of Yamanashi, Kofu, Japan

**Keywords:** Diabetes mellitus, Screening interval, Accuracy of testing

## Abstract

**Background:**

Guidelines for frequency of Type 2 diabetes mellitus (DM) screening remain unclear, with proposed screening intervals typically based on expert opinion. This study aims to demonstrate that HbA1c screening intervals may differ substantially when considering individual risk for diabetes.

**Methods:**

This was a multi-institutional retrospective open cohort study. Data were collected between April 1999 to March 2014 from one urban and one rural cohort in Japan. After categorization by age, we stratified individuals based on cardiovascular disease risk (Framingham 10-year cardiovascular risk score) and body mass index (BMI). We adapted a signal-to-noise method for distinguishing true HbA1c change from measurement error by constructing a linear random effect model to calculate signal and noise of HbA1c. Screening interval for HbA1c was defined as informative when the signal-to-noise ratio exceeded 1.

**Results:**

Among 96,456 healthy adults, 46,284 (48.0%) were male; age (range) and mean HbA1c (SD) were 48 (30–74) years old and 5.4 (0.4)%, respectively. As risk increased among those 30–44 years old, HbA1c screening intervals for detecting Type 2 DM consistently decreased: from 10.5 (BMI <18.5) to 2.4 (BMI > 30) years, and from 8.0 (Framingham Risk Score <10%) to 2.0 (Framingham Risk Score ≥20%) years. This trend was consistent in other age and risk groups as well; among obese 30–44 year olds, we found substantially shorter intervals compared to other groups.

**Conclusion:**

HbA1c screening intervals for identification of DM vary substantially by risk factors. Risk stratification should be applied when deciding an optimal HbA1c screening interval in the general population to minimize overdiagnosis and overtreatment.

## Background

Type 2 diabetes mellitus (DM) affects approximately 7.2 million adults in Japan, with a worldwide prevalence of 387 million in 2013 [1]. Its growing prevalence and strong association with a number of later complications, including cardiovascular events, has prompted the regular screening of healthy adults. However, screening guidelines for frequency of HbA1c testing remain unclear, and proposed screening intervals have been typically based on expert opinion.

In Japan, for example, a large majority of the population, including virtually all employed adults, receive free annual health checkups mandated by the 1972 Industrial Safety and Health Act, which typically include annual DM screening. In the United States, the American Diabetes Association recommends that screening for Type 2 DM in adults occur roughly every 3 years, though this is level IV evidence (expert opinion) [2–4]. In the UK, NICE guidelines recommend that general practitioners first utilize risk assessment tools such as the Cambridge diabetes risk score [5] or Leicester practice score [6] before measuring HbA1c, measuring HbA1c only if patients are found to be at high risk with subsequent re-screening every 3 years [7, 8]. Anecdotal data suggest, however, that clinicians provide overly frequent screening, often annually, for a large number of apparently healthy patients [9], despite existing data suggesting that this is not necessary [7, 10]. This may be especially problematic given that previous analyses of the characteristics of the HbA1c assay have demonstrated that the test possesses significant short-term variability, and that too-frequent testing may lead to diagnostic confusion [11].

The risk of developing Type 2 DM differs with age, obesity, and lack of physical activity [12]. A CDC report estimates an incidence of new onset diabetes of 7.8 per 1000 adults; however, this varies substantially from 3.6 cases for those under 44 years old to 12.0 for those from those 45–64 years old [13]. Similarly, a previous study reported that, in comparison with normal BMI, those with a BMI of 30–39.9 kg/m^2^ had an OR of 3.66 for being diagnosed with DM [14]. However, none of these previous studies have proposed different intervals for testing based on risk stratification [15, 16], despite suggesting that screening protocols tailored to specific at-risk patient populations are preferable.

In order to demonstrate that screening intervals may be substantially different when considering risk stratification, our study uses two clinically feasible risk stratification strategies, Framingham (10-year cardiovascular) risk [17] and BMI, to better define screening intervals while accounting for true change of HbA1c versus measurement error. We chose BMI as a basis of stratification as previous reports show different incidence rate of DM based on BMI classification [14]; we chose Framingham Risk Score as for stratification to reflect that DM is considered a clinical cardiovascular events -risk equivalent [18, 19]. We hypothesize that informative screening intervals for HbA1c will be shorter as individual risk increases.

## Methods

This was a retrospective open cohort study, combining an urban and rural population in Japan. The urban cohort was collected from St. Luke’s International Hospital Center for Preventive Medicine (Tokyo, Japan) between January 2005 and December 2014. Approximately 80% of participants were either employees or dependents of various companies and local government organizations in metropolitan Tokyo, health screening costs for whom were paid by the employer. The remaining 20% of participants were residents of Tokyo, independently registering and paying for the program. The rural cohort was collected from the Yamanashi Koseiren Health Care Center (Yamanashi, Japan) between April 1999 and March 2009 as part of a private health check-up service. Data in both cohorts included individuals presenting for health screening at least twice, with no previous history of Type 2 DM or cardiovascular events at first visit, and with complete data on risk equation covariates for Framingham Risk Score. Consistent with previous studies, patients were classified into 3 age groups (30–44, 45–59, and 60–74 years) [3, 15]. Clinically relevant demographic, historical, and lifestyle parameters were collected via a standardized questionnaire provided to all patients and reviewed by a trained healthcare provider. In addition to collecting physiometric data at the time of visit, HbA1c was collected as part of the standard serum testing panel from all patients at each visit. Each respective model was generated based on one of two risk stratification methods: (I) BMI classification (underweight, BMI <18.5; normal weight, BMI 18.6–24.9; overweight, BMI 25–29.9; and obese, BMI ≥30 [20, 21]); or (II) Framingham Risk Score for 10-year cardiovascular risk (low risk, <10%; moderate risk, 10% ≤ score < 20%; and high risk, ≥20%) [22, 23]. BMI and Framingham Risk Score parameters were collected during the routine health check up at the same time and in the same facility in which HbA1c was measured; the Framingham Risk Score has been previously validated in the Japanese population [24].

### Calculating signal and noise for laboratory testing

To distinguish true change in HbA1c progression, we adapted a statistical method for distinguishing the properties of tests from the variability of measurements; the methodology has been described in detail elsewhere [25, 26]. Briefly, linear random effect models with random intercept and random slope, adjusted for gender, age and BMI at first measurement of HbA1c as continuous value, were used to derive parameters describing HbA1c progression. These parameters include the long-term variability among individuals in the population (“signal”), as well as the short-term within-person variability (“noise”) (Appendix 5). In the random effect model, noise was obtained by calculating the variance of residuals between observed and model-generated HbA1c values. Signal was obtained by calculating, at each time point, the variance of random slope of each participant multiplied by each time point squared. Equations with detailed footnotes are shown in Table 1. We use the signal-to-noise ratio (SNR) as a quantitative marker to distinguish individuals with true HbA1c change from those with apparent change due to noise. Based on previous reports, we defined the minimal informative screening interval as the time at which the signal to noise ratio exceeds 1 [26, 27]. We calculated confidence intervals for these ratios through non-parametric bootstrapping (15,000 times).

**Table 1:** Equations of applied models

Random effect model:	*Y*_*ij*_ = *U*_*ij*_ + *ε*_*ij*_
Observed HbA1c:	*Y*_*ij*_
Noise:	*ε*_*ij*_
Signal:	*U*_*ij*_ = *α*_*ij*_ + *β*_*ij*_ * *T*
*α*_*i*_ ~ *N*(*α*_,_ *σ*_*a*_^*2*^) , *β*_*i*_ ~ *N*(*β*_,_ *σ*_*b*_^*2*^), *with covariance* (*α*_*i* ,_ *β*_*i*_) = *σ*_*ab*_ *ε*_*ij*_ ~ *N*(*0*_,_ *σ*_*w*_^*2*^)	

*Y* is the observed HbA1c, equal to the true change and the measurement error, ε. *U* is the true change in HbA1c for individual for individual _*i*_ at time _*j*_, α is the baseline HbA1c, β is the annual progression rate. *T* represents time since first measurement. The notation *~ N(x,y)* refers to a normal distribution with a mean *x* and a variance *y*, so the other main assumption of the model is normality in the distributions of α, β and ε. From this model, the short-term variability is equal to the variance of the measurement error (σ^2^w) whereas the long-term variability is equal to the variance of the annual progression rate (σ^2^β)

All models were fitted in Stata software version 12.1 (StataCorp LP, College Station, Texas, USA).

### Ethics, consent and permission

When patients presented to both medical facilities, they were provided with a document explaining that their anonymous data may be used for research purposes. While written informed consent was not provided, both institutions provided opt-out policy information in paper form and all patients had the opportunity to refuse use of their information from the electronic medical record. Ethical approval was obtained from two committees: the Research Ethics Committee of St. Luke’s International Hospital (approval code: 15-R044) as well as the Research Ethics Committee of University of Yamanashi (approval code: 1418).

## Results

**Fig. 1:**
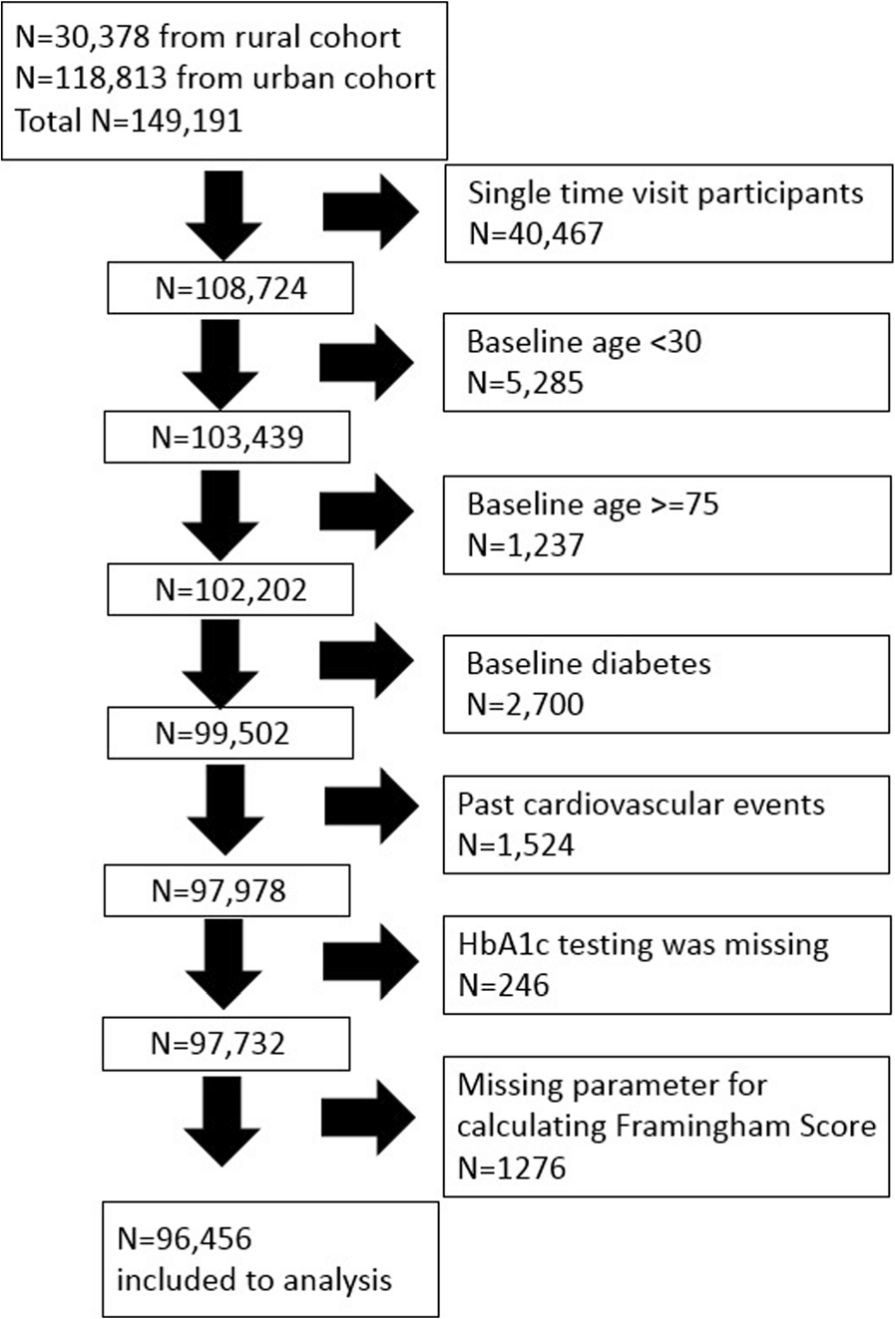
Study flow

Of the 149,191 adults in both cohorts, 96,456 healthy adults with an average age (SD) of 48.0 (10.6) years, comprising 46,284 (48.0%) males, were eligible for inclusion in this study. Exclusions are shown in the flow chart in Fig. 1. There were no clinically relevant differences between urban and rural cohorts in terms of HbA1c and BMI; mean HbA1c in the urban and rural cohorts was 5.5% (0.3) and 5.3% (0.4), respectively. Mean BMI was 22.3 (3.2) kg/m^2^ in the urban cohort and 22.7 (3.0) kg/m^2^ in the rural cohort. Mean (SD) age of the rural cohort was roughly 5 years older than that of the urban cohort at 51.2 (10.2) versus 46.5 (10.4) years, respectively. Mean (SD) Framingham Risk Score was slightly higher in rural than urban cohort, at 9.0% (0.08) and 6.0% (0.07), respectively. The proportion of current smokers in the rural cohort was twice that of the urban cohort. We merged the two cohorts into a single population for subsequent analyses to increase the generalizability of this study. Table 2 shows baseline characteristics for pooled data by age group. The majority of the cohort were of normal weight (BMI 18–24.9 kg/m^2^): 71.0% for ages 30–44, 72.4% for ages 45–59, and 74.0% for those 60–74 years old. Patients were similarly concentrated in the low risk (0–10%) group of Framingham Risk Score: 97.7% for those 30–44 years old, 73.3% for 45–59 years old, and 36.7% for those 60–74 years old.

**Table 2:** Baseline characteristics of study participants

Age between 30 and 44				
		Total	St. Luke’s	Yamanashi
Numbers		N = 41,400	N = 32,820	N = 8,580
Age		38.0 ± 3.9	37.8 ± 4.0	38.4 ± 3.8
Gender, male		19,572 (47.3%)	15,085 (46.0%)	4,487 (52.%)
Stratification				
10-year cardiovascular risk Framingham Risk Score	Lowest risk (0–10%)	40,434 (97.7%)	32,247 (98.3%)	8,187 (95.4%)
	Intermediate risk (10–20%)	859 (2.1%)	481 (1.5%)	378 (4.4%)
	High risk (over20%)	107 (0.3%)	92 (0.3%)	15 (0.2%)
BMI category	Underweight (bmi <18 · 5)	4,853 (11.7%)	3,950 (12.0%)	903 (10.5%)
	Normal (18 · 5 < =bmi < 25)	29,382 (71.0%)	23,365 (71.2%)	6,017 (70.1%)
	Overweight (25 < =bmi < 30)	6,199 (15.0%)	4,762 (14.5%)	1,437 (16.8%)
	Obese (30 < =bmi)	966 (2.3%)	743 (2.3%)	223 (2.6%)
Baseline HbA1c		5.3 ± 0.3	5.4 ± 0.3	5.2 ± 0.3
Baseline BMI		22.1 ± 3.3	22.0 ± 3.3	22.3 ± 3.4
Current smoker		8,562 (20.7%)	5,733 (17.5%)	2,829 (33.0%)
Age between 45 and 59				
		Total	St. Luke’s	Yamanashi
Numbers		N = 38,609	N = 24,539	N = 14,070
Age		51.6 ± 4.3	51.4 ± 4.3	51.9 ± 4.2
Gender, male		18,606 (48.2%)	12,162 (49.6%)	6,444 (45.8%)
Stratification				
10-year cardiovascular risk Framingham Risk Score	Lowest risk (0–10%)	28,298 (73.3%)	18,673 (76.1%)	9,625 (68.4%)
	Intermediate risk (10–20%)	8,293 (21.5%)	4,826 (19.7%)	3,467 (24.6%)
	High risk (over20%)	2,018 (5.2%)	1,040 (4.2%)	978 (7.0%)
BMI category	Underweight (bmi <18 · 5)	2,437 (6.3%)	1748 (7.1%)	689 (4.9%)
	Normal (18 · 5 < =bmi < 25)	27,960 (72.4%)	17,472 (71.2%)	10,488 (74.5%)
	Overweight (25 < =bmi < 30)	7,440 (19.3%)	4,767 (19.4%)	2,673 (19.0%)
	Obese (30 < =bmi)	772 (2.0%)	552 (2.2%)	220 (1.6%)
Baseline HbA1c		5.5 ± 0.4	5.5 ± 0.3	5.3 ± 0.4
Baseline BMI		22.7 ± 3.1	22.7 ± 3.2	22.8 ± 2.9
Current smoker		7,746 (20.1%)	4,225 (17.2%)	3,521 (25.0%)
Age between 60 and 74				
		Total	St. Luke’s	Yamanashi
		N = 16,447	N = 8,801	N = 7,646
Age		64.6 ± 3.8	65.0 ± 3.9	64.2 ± 3.7
Gender, male		8,106 (49.3%)	4,513 (51.3%)	3,593 (47.0%)
Stratification				
10-year cardiovascular risk Framingham Risk Score	Lowest risk (0–10%)	6,041 (36.7%)	3,341 (37.9%)	2,700 (35.3%)
	Intermediate risk (10–20%)	6,276 (38.2%)	3,356 (38.1%)	2,920 (38.2%)
	High risk (over20%)	4,130 (25.1%)	2,104 (23.9%)	2,026 (26.5%)
BMI category	Underweight (bmi <18 · 5)	933 (5.7%)	573 (6.5%)	360 (4.7%)
	Normal (18 · 5 < =bmi < 25)	12,164 (74.0%)	6,533 (74.2%)	5,631 (73.4%)
	Overweight (25 < =bmi < 30)	3,164 (19.2%)	1,606 (18.2%)	1,558 (20.4%)
	Obese (30 < =bmi)	186 (1.1%)	89 (1.0%)	97 (1.3%)
Baseline HbA1c		5.6 ± 0.4	5.6 ± 0.3	5.4 ± 0.4
Baseline BMI		22.8 ± 2.8	22.6 ± 2.9	23.0 ± 2.8
Current smoker		1,995 (12.1%)	855 (9.7%)	1,140 (14.9%)

### Screening and BMI

**Fig. 2:**
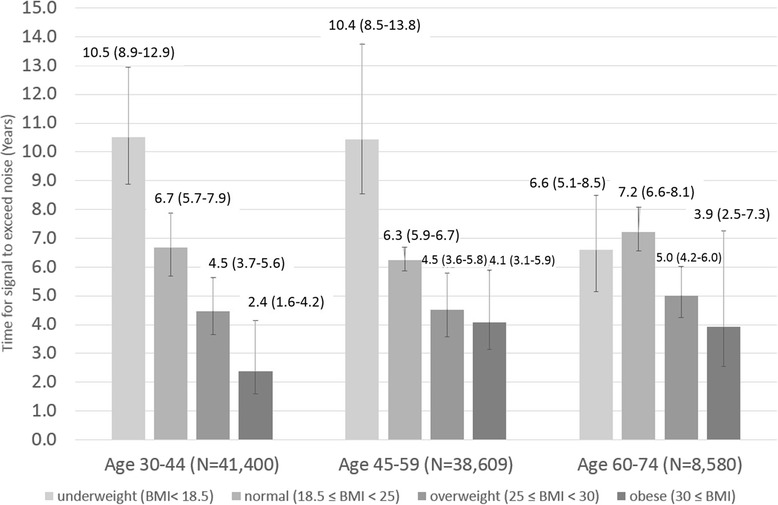
DM screening intervals for HbA1c screening test by BMI stratification (years). (−) indicates 95% CI of screening interval for HbA1c screening test calculated by non-parametric 15000 times bootstrapping simulations

Figure 2 show the time interval at which the signal exceeded noise for each BMI stratification by age group. For those aged 30–59 years old, the DM screening interval for HbA1c decreased as BMI increased. In underweight and normal weight individuals 60–74 years old, screening intervals were similar. In all age groups, underweight and normal weight individuals appeared to warrant less HbA1c screening compared to those in the heavier group. Obesity in the 30–44 year old group was associated with substantially shorter intervals compared to other age groups.

### Screening and cardiovascular risk

**Fig. 3:**
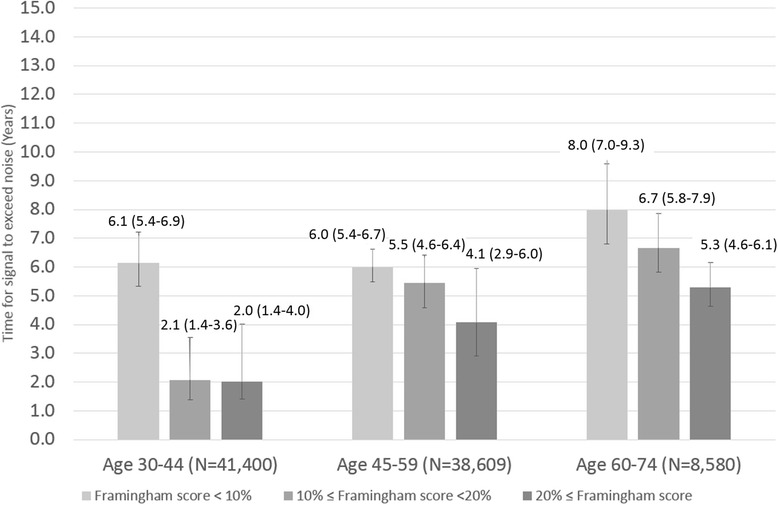
DM screening intervals for Hba1c screening test by Framingham Risk Score stratification (years). (−) indicates 95% CI of screening interval for HbA1c screening test calculated by non-parametric 15,000 times bootstrapping simulations

Figure 3 shows the time at which the signal exceeded noise, stratified by Framingham Risk Score and age. For all age groups, DM screening intervals decreased as Framingham Risk Score increased. Similar to BMI stratification, the highest Framingham risk group in those 30–44 years old demonstrated a much shorter interval than those in other age groups. These results were consistent even after analyzing data in each cohort independently (Appendix 1, 2, 3 and 4). We found that informative intervals were, predictably, shorter in the high DM risk group compared to low risk group (Appendix 6) [28].

## Discussion

Meaningful screening intervals varied substantially by BMI, ranging from as long as 10.5 (8.9–12.9) years to as short as 2.4 (1.6–4.2) years. When Framingham cardiovascular risk was used for stratification, monitoring intervals were similarly varied, from 8.0 (7.0–9.3) to 2.0 (1.4–4.0) years. Regardless of the stratification method employed, analysis of higher risk groups consistently resulted in shorter screening intervals compared to those at lower risk. While existing guidelines [7, 10] do mention screening intervals, these have been typically based on a combination of previous studies [15, 16] and expert opinion, none of which have fully considered risk as a criteria for screening. An evidence-based optimal screening interval has not yet been fully explored; our data suggest that consideration of at least some baseline risk characteristics is warranted. A 3–5 year monitoring interval suggested by Kahn et al., while providing good evidence for cost-effectiveness, does not apply to those over 45 years old and, more importantly, may not adequately account for varying baseline risk in the adult population [15]. Further highlighting the possibility that screening unselected populations for DM may not be efficacious, Simmons et al. reported finding no association between screening and reduction in all-cause, cardiovascular, or diabetes-related mortality within 10 years [29, 30], a conclusion that was corroborated in a recent systematic review by the Agency for Healthcare Research and Quality [31]. Our findings suggest that low risk individuals with a long screening interval, such as the well elderly, may not warrant screening.

Despite a growing body of evidence that population-level DM screening may not be useful, we anticipate that it will remain unpalatable from a societal perspective to do away with screening entirely, especially in light of recent reports of the alarmingly high prevalence of DM and its risk factors, including obesity [32]. As demonstrated by the substantial heterogeneity of SNR between risk groups, our data suggest that optimal screening, rather than following an all-or-nothing approach, should be tailored to match broad risk profiles, which can be easily assessed at clinic visits, to improve screening accuracy. Based on actual physician practice, the previously reported intervals of approximately 3–5 years may represent reasonable minimal thresholds of re-screening; however, they appear to be inadequate representations of re-screening ceilings, which may be substantially longer in low-risk patients. Future studies should address cost-effectiveness after considering risk stratification in the assessment of screening intervals.

This study has some limitations. We relied on self-reports for some clinical criteria, such as use of DM medication, DM and cardiovascular event history, as well as current smoking status for calculating Framingham Risk Scores. Differences in data collection technique may have existed between the two cohorts: participants in the urban cohort received a personalized interview by a trained preventive health nurse, in order to maximize accuracy of data, while there was no nurse interview system in the rural cohort. Despite this, clinically relevant differences were not seen between the two cohorts. Second, this was a multi-institutional open cohort study of data collected from urban and rural Japan. Generalizability to other countries warrants further exploration. Finally, under current Japanese occupational health law, contents of health exams may differ based on employee age; those under 39 or over 75 years old are not required to have blood tests, thus raising the possibility of selection bias via missing data, especially for those 30–39 years old. However, most companies choose to include screening blood tests for all employees; in the urban cohort, only 0.03% of participants failed to have a blood test panel.

## Conclusion

When stratifying by risk for DM development, HbA1c screening intervals varied substantially. Taking into account differences of true HbA1c change, to avoid overdiagnosis and overtreatment, risk stratification should be applied when deciding an optimal HbA1c screening interval in a specific population. Annual screening appears to be unwarranted in any risk group, while those at low risk may not warrant re-screening for a decade or longer.

## Figures and Tables

**Fig. 4:**
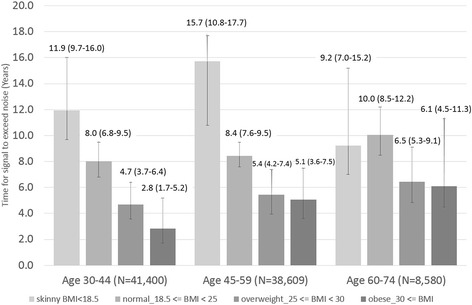
DM screening intervals for HbA1c screening test in the urban cohort by BMI stratification. Description of data: Figure as indicated in title

**Fig. 5:**
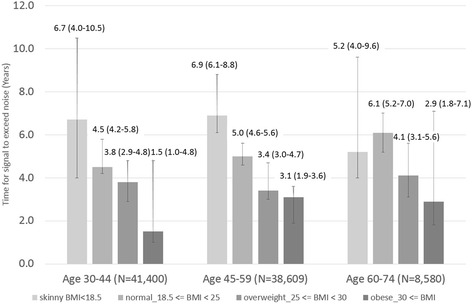
DM screening intervals for HbA1c screening test in the rural cohort by BMI stratification. Description of data: Figure as indicated in title

**Fig. 6:**
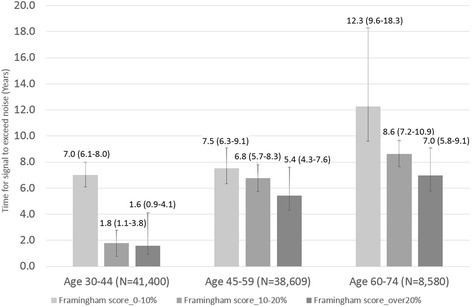
DM screening intervals for HbA1c screening test in the urban cohort by Framingham Risk Score stratification. Description of data: Figure as indicated in title

**Fig. 7:**
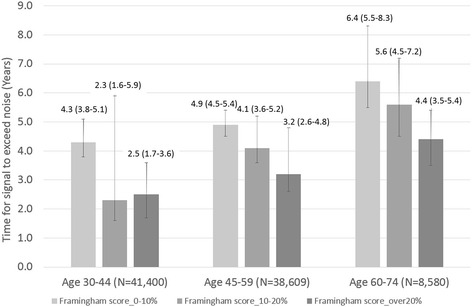
DM screening intervals for HbA1c screening test in the rural cohort by Framingham Risk Score stratification. Description of data: Figure as indicated in title

## Appendix 5

**Table 3:** Signal and noise of HbA1c, time when signal > noise (years) by risk group

Age category	Stratification		HbA1c signal change per year (%)^a^	HbA1c noise (%)^a^	Time when Signal > Noise
30–44	BMI	Underweight	0.02	0.18	10.5
		Normal	0.03	0.18	6.7
		Overweight	0.05	0.22	4.5
		Obese	0.13	0.30	2.4
	Framingham Risk Score	0–10%	0.03	0.19	6.1
		10–20%	0.11	0.23	2.1
		Over 20%	0.10	0.20	2.0
45–59	BMI	Underweight	0.02	0.18	10.4
		Normal	0.03	0.18	6.3
		Overweight	0.05	0.22	4.5
		obese	0.08	0.31	4.1
	Framingham Risk Score	0–10%	0.03	0.18	6.0
		10–20%	0.04	0.21	5.5
		Over 20%	0.06	0.25	4.1
60–74	BMI	Underweight	0.03	0.17	6.6
		Normal	0.03	0.18	7.2
		overweight	0.04	0.21	5.0
		Obese	0.06	0.23	3.9
	Framingham Risk Score	0–10%	0.02	0.17	8.0
		10–20%	0.03	0.19	6.7
		Over 20%	0.04	0.21	5.3

^a^Signal defined as variance of random slope; noise defined as variance of residual between model-generated and observed HbA1c. Values represent square root of above measures to obtain HbA1c as %

## Appendix 6

**Table 4:** DM screening intervals for HbA1c screening test by DM score (years)

	Age 30–44	Age 45–59	Age 60–74
High DM risk^a^	4.8	5.4	5.5
Low DM risk	8.2	7.2	7.3

^a^Risk score calculated based on Nanri et al. [28]; cut-off point modified from 10 to 9 due to omission of waist circumference

## Abbreviation

BMI: Body mass index
DM: Type 2 diabetes mellitus
HbA1c: Hemoglobin A1c
OR: Odds ratio
SD: Standard deviation
SNR: Signal to noise ratio
